# Ocular manifestations in Ehlers-Danlos syndrome

**DOI:** 10.1038/s41433-025-03787-1

**Published:** 2025-04-10

**Authors:** Sonia B. Kim, Jacqueline K. Shaia, David C. Kaelber, Rishi P. Singh, Katherine E. Talcott

**Affiliations:** 1 Case Western Reserve University School of Medicine, Cleveland, OH USA; 2 Center for Ophthalmic Bioinformatics, Cole Eye Institute, Cleveland Clinic, Cleveland, OH USA; 3 The Center for Clinical Informatics Research and Education, The MetroHealth System and Departments of Internal Medicine, Pediatrics, and Population and Quantitative Health Sciences, Case Western Reserve University, Cleveland, OH USA; 4 Cleveland Clinic Martin Hospitals, Cleveland Clinic Florida, Stuart, FL USA; 5 Cleveland Clinic Cole Eye Institute, Cleveland, OH USA; 6 Cleveland Clinic Lerner College of Medicine of Case Western Reserve University, Cleveland, OH USA

**Keywords:** Epidemiology, Retinal diseases

## Abstract

**Background/Objective:**

To provide a large-scale analysis on the demographics and ocular comorbidities in Ehlers-Danlos Syndrome (EDS) patients in the US.

**Subjects/Methods:**

This is an exploratory cross-sectional study comparing medical records of EDS patients to the general population on demographic variables and ICD-10 ocular diagnoses. A research platform with de-identified EHR data of over 99 million patients across 60 healthcare organizations was utilized. Groups were stratified by 30-year age groups. Patients aged 0–61+ with an ICD-10 diagnosis of EDS (76,526), the general platform population aged 0–61+ (99,836,639), and patients with a concurrent ICD-10 ocular diagnosis were queried to determine the prevalence of EDS across demographic variables, ocular disease, and odds of ocular disease. Statistical analysis was conducted using Microsoft Excel and R studio, using *p* < 0.01 and 95% confidence intervals (CI).

**Results:**

An EDS diagnosis was most prevalent in white females aged 0–30 years old (259.6 per 100,000). The majority of ocular diagnoses were more prevalent in the 0–60-year-old EDS population compared to the general population including myopia (5227.0 per 100,000) and dry eye (4211.6 per 100,000). Overall, diagnoses of angioid streaks (POR 18.72, 95% CI 10.32, 33.94) and idiopathic intracranial hypertension (IIH) (POR 18.43, 95% CI 17.51, 19.39) showed the highest increased odds in patients with EDS while significantly decreased odds were shown for type 2 diabetic retinopathy, age-related macular degeneration, and retinal vein occlusion.

**Conclusions:**

EDS was associated with increased odds of having a concurrent ocular pathology, suggesting that, upon diagnosis of EDS, referral to ophthalmology may be valuable.

## Introduction

Ehlers-Danlos syndrome (EDS) is a rare genetic disorder of collagen and extracellular matrix proteins. It has a reported prevalence between 1 in 5000 and 1 in 100 000 [1, 2]. This value is likely an underestimation due to the wide spectrum of clinical presentations in EDS, attributed to variants in 20 genes [3, 4]. The variable expressivity of these genes creates 13 different subtypes of EDS, most recently classified by the International EDS Consortium in 2017 [5]. Because the implicated genes encode for fibrillar collagen and collagen modifying enzymes, the manifestations of the EDS subtypes have common features: joint hypermobility, skin hyperelasticity and hyperextensibility, abnormal wound healing, and tissue fragility [3, 6–9]. The ubiquitous nature of connective tissue entails that EDS affects nearly every organ system in the body. Thus, although the most widely recognized systems in EDS are the musculoskeletal, cardiovascular, gastrointestinal, and dermatological systems, a diagnosis of EDS warrants an extensive work-up to assess the integrity of connective tissue elsewhere [3, 10–13].

Little is known regarding the ocular manifestations of EDS with most studies being case studies or series. Perez-Roustit et al. published a series of 21 patients with EDS and reported ophthalmological signs in 90% of their cohort including dry eye, blue sclera, and high myopia [14]. Other studies detail findings of irregular astigmatism, conjunctivochalasis, and ectopia lentis [15–17]. One prospective, cross-sectional study of 44 eyes with EDS of the hypermobile subtype reported xerophthalmia, steeper corneas, myopia, lens opacity, and vitreous abnormalities [18]. To the best of our knowledge, the average sample sizes of studies detailing ocular features in EDS patients are less than 100 and there was a lack of consensus in the ocular findings reported [19, 20]. Despite the spectrum of ocular findings in EDS, the pathogenesis of each condition is likely rooted in abnormal collagen fibrillogenesis that affects the collagenous structures of the eye.

From an ophthalmic surgery standpoint, a case study of 467 EDS patients found that 24% of the cohort required ophthalmic surgery, including but not limited to strabismus, refractive, retinal, and cataract surgery [21]. Nearly 50% of these patients experienced at least one complication. Awareness of this post-op complication rate could shape surgical decision making, yet 76% of those who underwent surgery were not yet diagnosed with EDS. Providing additional information in the literature about EDS ocular phenotypes could help surgeons screen patients prospectively for EDS, and also expedite a diagnosis that may affect surgical planning.

In the setting of a rare disease such as EDS, it is furthermore difficult to diagnose the relevant subtype of disease as clinical criteria and genetic markers continue to evolve [22–24]. Not only does this pose a difficulty for ophthalmologists when evaluating EDS patients, but it also generates uncertainty in counselling patients on what their EDS diagnosis means for their eye health. The use of aggregated electronic health records (EHR) enables an unprecedented ability to analyse the largest group of EDS patients to date. Thus, the primary purpose of this exploratory analysis was to provide updated information on EDS patient demographics and ocular comorbidities.

## Materials/subjects and methods

This retrospective, cross-sectional study was conducted using the TriNetX US Collaborative Network (TriNetX, LLC (www.trinetx.com)), a federated health research platform that aggregates de-identified EHR data of over 99 million patients across 60 healthcare organizations (HCOs). TriNetX, LLC is compliant with the Health Insurance Portability and Accountability Act (HIPAA), the US federal law which protects the privacy and security of healthcare data, and any additional data privacy regulations applicable to the contributing HCO. The process by which the data is de-identified is attested to through a formal determination by a qualified expert as defined in Section §164.514(b)(1) of the HIPAA Privacy Rule. Because this study used only de-identified patient records, this study was deemed exempt by the Western Institutional Review Board.

Data was collected on July 1, 2024 with a restricted timeframe of 1/1/2012-5/1/2024. Additional data was collected on February 11, 2025 to address reviewer comments. All encounter diagnosis data was extracted using International Statistical Classification of Diseases and Related Health Problems, Ninth/Tenth Revision (ICD-9/10) codes as the platform automatically groups these together. Of note, data reporting throughout this manuscript is not a measure of the true study populations as the platform relies on existing data within EHR systems. Thus, in this paper, an EDS patient is defined as a patient who has a recorded ICD-10 diagnosis of EDS, the general population is defined as any individual with a visit in the EHR irrespective of an associated ICD-10 diagnosis, and an ocular condition is defined as a recorded ICD-10 diagnosis of this condition.

The first aim of this study was to highlight how the EDS patient population may differ from the general population by demographic factors of age, gender, and race. The EDS patient cohort was defined by any ICD-10 encounter diagnosis code Q79.6 and compared to the entire TriNetX population in the US Analytic Network. Each cohort was stratified by 30-year age cohorts to capture nuances in this population who is typically in adolescence at time of first diagnosis and has a significantly shortened life expectancy at 48 years [25, 26]. Within each cohort, the mean age of the study group (and associated standard deviation) was collected to assist in drawing comparative conclusions from the data. Within each age group, gender and race/ethnicity stratifications were also added.

Secondarily, this study aimed to understand the recorded prevalence of ocular diagnoses in EDS patients compared to the general population through obtaining the platform’s prevalence data on multiple ocular conditions. The equation used for the prevalence of an ocular condition in EDS per 100000 was: $\frac{{{Prevalence\; of\; ocular\; disease}}_{{in\; EDS}}}{{Total\; EDS\; population}}x100000.$ Conditions commonly reported in the EDS literature, conditions with increased risk with cardiovascular disease, and other common retinal conditions were included. All ICD-10 encounter diagnoses codes for these conditions are listed in Table 1. Age was considered as a confounding variable due to the decreased life expectancy of the EDS group, and the increased average age of diagnosis for several conditions examined. To account for this, the prevalence of these conditions was obtained at the same 30-year age stratification rate. The platform automatically rounds any counts 1–10 to protect patient privacy. Therefore, ocular conditions that resulted in less than or equal to 10 patient cases in the platform were omitted from calculations and figures to prevent presentation of misleading data.

**Table 1:** ICD-10 encounter diagnoses codes used in TriNetX queries for ocular conditions.

Ocular conditions	ICD-10 code
Retinal tear/detachment	H33
Myopia	H52.1
Degenerative myopia	H44.2
Aphakia	H27.0
Pseudophakia	Z96.1
Lens subluxation	H27.11
Glaucoma	H40-H42
IIH	G93.2
Diabetic retinopathy (T1DM)	E10.3
Diabetic retinopathy (T2DM)	E11.3
Age-related macular degeneration (neovascular)	H35.31
Age-related macular degeneration (non-neovascular)	H35.32
Choroidal neovascularization	H35.059
Macular cyst / hole / pseudohole	H35.34
Retinopathy of prematurity	H35.1
Peripheral retinal degeneration	H35.4
Angoid streaks	H35.33
Dry eye syndrome	H04.12
Carotid cavernous fistula	I67.1
Retinal artery occlusion (central, branch)	H34.10, H34.23
Retinal vein occlusion (central, branch)	H34.81, H34.83
Hypertensive retinopathy	H35.03
Ischemic optic neuropathy	H47.01

To assess whether EDS patients were at increased odds for any of the studied ocular conditions, a contingency table was used for the prevalence odds ratio (POR) calculation. With age stratification, some ocular conditions too rare to stratify by age were rounded by the platform, and thus excluded for the analysis. PORs and their 95% confidence intervals (CI) were calculated using Microsoft Excel and R Studio. Forest plots were created in R Studio. *P* values were calculated for all prevalence estimates using a chi-squared 2-sided test. A significance threshold of 0.01 or less was used. STROBE guidelines were followed in this study and writeup.

## Results

At the time of data collection, the total number of records with an EDS diagnosis was 76526 and the total data population in the US Collaborative Network was 99 836 639.

### EDS patient demographics

Across each race stratification, females had a greater prevalence of EDS diagnosis compared to men (Table 2). Records of an EDS diagnosis were most prevalent in white females 30 years or younger with a prevalence of 259.6 per 100,000. In the 30-year age cohorts, the total prevalence of EDS records per 100,000 was respectively 110.8, 93.8, and then a steep drop in the 61+ cohort to 17.1. When stratifying by gender and age, the recorded EDS prevalence for females aged 0–30 and 31–60 were comparable at respectively 167.3 and 150.0. There was a drop in the prevalence of females 61+ years old diagnosed with EDS at 31.7 per 100,000. This trend of decreased prevalence of an EDS diagnosis at 61+ years old was seen in all races/ethnicities when female gender was isolated. Although male patients had a much smaller EDS diagnosis prevalence compared to females, an EDS diagnosis was also most prevalent in males aged 0–30 across all races investigated (Table 2).

**Table 2:** Stratification of EDS patients by 30-year age cohorts, gender, and race.

	Age Cohort								
	0–30			31–60			61 +		
	Prevalence of EDS per 100,000 (n)	Total Population (n)	*P* value	Prevalence of EDS per 100,000 (n)	Total Population (n)	*P* value	Prevalence of EDS per 100,000 (n)	Total Population (n)	*P* value
**Total**	110.8 (36,051)	32,526,747	<0.001	93.8 (34,187)	36,462,584	<0.001	17.1 (5273)	30,847,308	<0.001
**Female**	167.3 (27,150)	16,231,801	<0.001	150.0 (29,668)	19,774,595	<0.001	31.7 (5082)	16,017,901	<0.001
**Male**	54.1 (8382)	15,487,207	<0.001	23.5 (3674)	15,639,479	<0.001	7.2 (978)	13,673,772	<0.001
**White Female**	259.6 (22,310)	8,592,592	<0.001	224.8 (25,031)	11,137,082	<0.001	42.6 (4452)	10,461,665	<0.001
**White Male**	82.9 (6820)	8,224,301	<0.001	33.1 (2945)	8,904,254	<0.001	9.0 (820)	9,087,338	<0.001
**Black Female**	26.9 (693)	2,579,233	<0.001	23.5 (707)	3,008,525	<0.001	6.5 (116)	1,796,675	<0.001
**Black Male**	8.6 (210)	2,449,689	<0.001	5.3 (123)	2,301,449	<0.001	2.6 (35)	1,347,358	<0.001
**Hispanic Female**	55.9 (1478)	2,643,493	<0.001	48.9 (1053)	2,154,312	<0.001	9.3 (76)	820,367	<0.001
**Hispanic Male**	21.8 (559)	2,563,498	<0.001	8.7 (142)	1,623,389	<0.001	4.9 (32)	650,851	<0.001
**Asian Female**	51.1 (315)	617,003	<0.001	35.2 (303)	860,041	<0.001	6.1 (31)	507,957	<0.001
**Asian Male**	14.0 (85)	606,368	<0.001	10.7 (63)	586,240	<0.001	3.4 (14)	406,148	<0.001

### EDS and ocular conditions

Table 3 showcased the overall prevalence of patients with ICD-10 encounter diagnoses for EDS and ocular conditions, and the prevalence within the 30-year age cohorts. The mean age of patients diagnosed with EDS (22 ± 6 years of age) was older than the platform’s general population (17 ± 8 years of age) only for the 0–30 age-cohort. Overall, 9/23 (40%) included ocular diagnoses were significantly more prevalent in EDS diagnosed patients compared to the total population. Specifically, patients with recorded diagnoses of myopia and dry eye syndrome had the highest prevalence per 100,000 at 5227.0 and 4211.6 respectively. Notably, diagnoses of idiopathic intracranial hypertension (IIH) and glaucoma demonstrated marked prevalence per 100,000 at 1995.4 and 1532.8. ICD-10 encounter diagnoses for several ocular conditions were found to have significantly lower prevalences among patients diagnosed with EDS than the overall population including: diabetic retinopathy (DR) secondary to type 2 diabetes mellitus (T2 DR), non-neovascular age-related macular degeneration (AMD), and neovascular AMD (Table 3).

**Table 3:** Comparison of the prevalence of ocular conditions in EDS versus the total population stratified by 30-year age cohorts; NA= Conditions with less than 10 patient cases in TriNetX.

Age cohort												
	0–30			31–60			61 +			Overall total		
	EDS	Total Population	*P* value	EDS	Total Population	*P* value	EDS	Total Population	*P* value	EDS	Total Population	*P* value
Median age (SD)	22 (6)	17 (8)		43 (8)	45 (9)		70 (7)	75 (9)		35 (16)	46 (25)	
	**Prevalence of Ocular Condition in EDS per 100,000 (n)**	**Prevalence of Ocular Condition in Total Population per 100,000 (n)**		**Prevalence of Ocular Condition in EDS per 100,000 (n)**	**Prevalence of Ocular Condition in Total Population per 100,000 (n)**		**Prevalence of Ocular Condition in EDS per 100,000 (n)**	**Prevalence of Ocular Condition in Total Population per 100,000 (n)**		**Prevalence of Ocular Condition in EDS per 100,000 (n)**	**Prevalence of Ocular Condition in Total Population per 100,000 (n)**	
Retinal tear/detachment	58.8 (45)	16.9 (16,879)	<0.001	186.9 (143)	73.0 (72,887)	<0.001	147.7 (113)	160.8 (160,572)	0.39	393.3 (301)	250.7 (250,338)	<0.001
Myopia	2301.2 (1761)	385.6 (384,964)	<0.001	2444.9 (1871)	469.3 (468,558)	<0.001	480.9 (368)	399.4 (398,733)	<0.001	5227.0 (4000)	1254.3 (1,252,255)	<0.001
Degenerative myopia	43.1 (33)	11.8 (11,758)	<0.001	96.7 (74)	15.9 (15,911)	<0.001	36.6 (28)	23.1 (23,042)	0.019	176.4 (135)	50.8 (50,711)	<0.001
Aphakia	19.6 (15)	8.2 (8174)	<0.001	19.6 (15)	7.5 (7504)	<0.001	NA	19.0 (18,991)	NA	48.3 (37)	34.7 (34,669)	0.1
Pseudophakia	27.4 (21)	9.8 (9797)	<0.001	111.1 (85)	46.4 (46,296)	<0.001	379.0 (290)	621.9 (620,848)	<0.001	517.5 (396)	678.0 (676,941)	<0.001
Lens subluxation	15.7 (12)	1.3 (1.262)	<0.001	NA	1.9 (1920)	NA	NA	5.7 (5716)	NA	15.7 (12)	8.9 (8,898)	0.1
Glaucoma	260.0 (199)	49.1 (49,008)	<0.001	734.4 (562)	245.7(245,314)	<0.001	538.4 (412)	1008.3 (1,006,629)	<0.001	1532.8 (1173)	1303.1 (1,300,951)	<0.001
IIH	670.4 (513)	28.3 (28,214)	<0.001	1263.6 (967)	64.6 (64,541)	<0.001	61.4 (47)	17.6 (17,552)	<0.001	1995.4 (1527)	110.5 (110,307)	<0.001
Diabetic retinopathy (T1DM)	NA	4.1 (4056)	NA	45.7 (35)	36.5 (36,438)	0.21	NA	34.9 (34,851)	NA	68.0 (52)	75.5 (75,345)	0.5
Diabetic retinopathy (T2DM)	NA	2.9 (2912)	NA	113.7 (87)	136.4 (136,210)	0.098	133.3 (102)	434.4 (433,677)	<0.001	249.6 (191)	573.7 (572,799)	<0.001
Age-related macular degeneration (neovascular)	NA	0.3 (307)	NA	17.0 (13)	4.6 (4605)	<0.001	111.1 (85)	201.6 (201,236)	<0.001	129.4 (99)	206.5 (206,148)	<0.001
Age-related macular degeneration (non-neovascular)	NA	0.2 (157)	NA	19.6 (15)	2.1 (2051)	<0.001	37.9 (29)	94.0 (93,873)	<0.001	57.5 (44)	96.2 (96,081)	<0.001
Choroidal neovascularization	NA	0.5 (462)	NA	NA	2.1 (2061)	NA	NA	13.0 (12,947)	NA	20.9 (16)	15.5 (15,470)	0.3
Macular cyst / hole / pseudohole	NA	1.5 (1451)	NA	20.9 (16)	8.4 (8366)	<0.001	45.7 (35)	55.3 (55,225)	0.29	69.3 (53)	65.1 (65,042)	0.7
Retinopathy of prematurity	35.3 (27)	63.1 (63,034)	0.0027	NA	1.4 (1395)	NA	NA	1.3 (1340)	NA	43.1 (33)	65.9 (65,769)	0.0
Peripheral retinal degeneration	61.4 (47)	9.6 (9567)	< 0.001	201.2 (154)	41.2 (41,138)	<0.001	70.6 (54)	64.7 (64,639)	0.57	333.2 (255)	115.5 (115,344)	<0.001
Angoid streaks	NA	0.0 (41)	NA	NA	0.2 (266)	NA	NA	0.5 (501)	NA	14.4 (11)	0.8 (768)	<0.001
Dry eye syndrome	761.8 (583)	55.6 (55,538)	<0.001	2485.4 (1901)	329.0 (328,460)	<0.001	964.4 (738)	746.8 (745,593)	<0.001	4211.6 (3223)	1131.4 (1,129,591)	<0.001
Carotid cavernous fistula	137.2 (105)	5.5 (5518)	<0.001	530.5 (406)	62.7 (62,613)	<0.001	198.6 (152)	151.1 (150,865)	<0.001	866.4 (663)	219.4 (218,996)	<0.001
Retinal artery occlusion	NA	0.3 (307)	NA	NA	3.5 (3489)	NA	NA	18.0 (17,965)	NA	18.3 (14)	21.8 (21,761)	0.6
Retinal vein occlusion	NA	0.6 (584)	NA	22.2 (17)	8.8 (8791)	<0.001	28.7 (22)	51.0 (50,871)	0.0082	53.6 (41)	67.1 (67,008)	0.2
Hypertensive retinopathy	NA	0.7 (715)	NA	17.0 (13)	18.3 (18,257)	0.89	23.5 (18)	65.9 (65,755)	<0.001	40.5 (31)	81.1 (81, 013)	<0.001
Ischemic optic neuropathy	NA	0.8 (750)	NA	15.7 (12)	6.7 (6688)	0.0049	NA	26.0 (25,943)	NA	35.3 (27)	33.4 (33,381)	0.9

In the 0–30 year age cohort, 11/12 (92%) ocular diagnoses were significantly more prevalent in patients diagnosed with EDS compared to the overall population. Those ICD-10 encounter diagnosis codes that remained more prevalent in patients diagnosed with EDS were myopia (prevalence in EDS per 100,000: 2301.2), dry eye syndrome (761.8), IIH (670.4), and glaucoma (260.0). In the 31–60 year age cohort, 15/18 (83%) ocular diagnoses were significantly more prevalent in patients diagnosed with EDS compared to the overall population. ICD-10 encounter diagnoses with markedly increased prevalence in this cohort were similarly myopia (2444.9), dry eye syndrome (2485.4), IIH (1263.6), and glaucoma (734.4). Lastly, in the 61+ year old cohort, 4/15 (27%) ocular diagnoses were significantly more prevalent in patients diagnosed with EDS compared to the overall population. In this older group of patients, the most prevalent ICD-10 encounter diagnosis was dry eye syndrome (964.4), followed by glaucoma (538.4) and myopia (480.9).

**Fig. 1:**
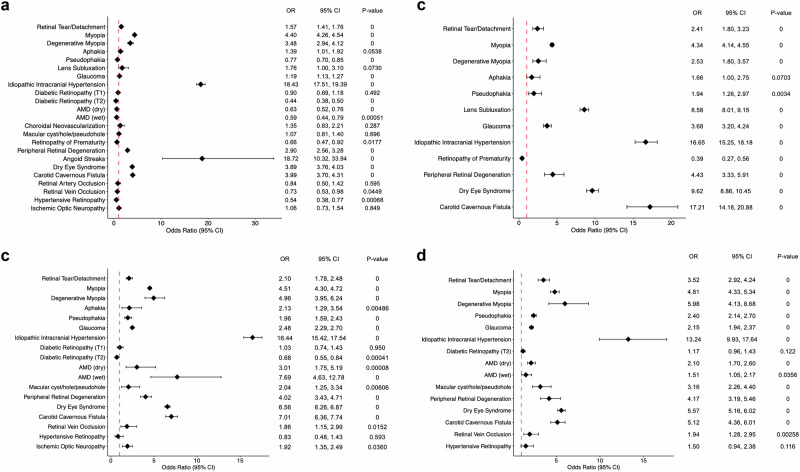
Forest plots depicting odds of ocular diagnoses in various EDS patient age groups. **a** Forest plot depicting prevalence odds ratios (PORs) of various ocular diagnoses in the overall EDS population. **b** Forest plot depicting prevalence odds ratios (PORs) of various ocular diagnoses in the EDS patients 0–30 years old. **c** Forest plot depicting prevalence odds ratios (PORs) of various ocular diagnoses in the EDS patients 31–60 years old. **d** Forest plot depicting prevalence odds ratios (PORs) of various ocular diagnoses in the EDS patients 61+ years old.

After calculating the PORs between patients diagnosed with EDS and the overall population, 40% of ocular diagnoses continued to be significantly increased in EDS patients. Notably, the highest PORs were for recorded ICD-10 diagnoses of angioid streaks (POR 18.72, 95% CI 10.32, 33.94, *p* value 0) and IIH (POR 18.43, 95% CI 17.51, 19.39, *p* value 0). Other ocular diagnoses with increased PORs in EDS records were myopia (POR 4.40, 95% CI 4.26, 4.54, *p* value 0), degenerative myopia (POR 3.48, 95% CI 2.94, 4.12, *p* value 0), dry eye syndrome (POR 3.89, 95% CI 3.76, 4.03, *p* value 0), and carotid cavernous fistula (POR 3.99, 95% CI 3.70, 4.31, *p* value 0). On the other hand, patients diagnosed with EDS were at significantly decreased odds for multiple ocular diagnosis including T2 DR and AMD (*p* < 0.01) (Fig. 1a).

When stratifying by age, only ocular diagnoses with >10 records were included in the analysis. In the 0–30 age cohort, the POR was significantly increased for 10/12 conditions (83%) and significantly decreased for 1/12 conditions (8%). In 0–30-year-old patients diagnosed with EDS, the ocular diagnoses with markedly increased odds were carotid cavernous fistula (POR 17.21, 95% CI 14.18, 20.88, *p* value 0) and IIH (POR 16.65, 95% CI 15.25, 18.18, *p* value 0). ROP showed decreased odds in patients diagnosed with EDS 0–30 years old (POR 0.39, 95% CI 0.27, 0.56, *p* value 0). In the 31–60 age cohort, EDS records were at significantly increased odds of having a recorded diagnosis for 72% of the included conditions, while having significantly decreased odds of 1/18 (6%) of the diagnoses. The ICD-10 diagnosis for IIH showed the highest increased odds (POR 16.44, 95% CI 15.42, 17.54, *p* value 0) but the ICD-10 diagnosis for T2 DR showed significantly decreased odds (POR 0.68, 95% CI 0.55, 0.84, *p* value 0.00041). Lastly, in the 61+ age cohort, 12/15 (80%) of ocular diagnoses were significantly increased in patients diagnosed with EDS, with the ICD-10 diagnosis for IIH consistently showing the highest increased odds. All data corresponding to the PORs, CIs, and *p* values of ocular ICD-10 encounter diagnoses in EDS by age can be found in Fig. 1b–d.

## Discussion

This exploratory study used the TriNetX platform to examine the demographics of EDS and prevalence of ocular conditions in patients with recorded diagnoses of EDS, distributed across the age cohorts 0–30, 31–60, and 61 + . We found an EDS diagnosis was most prevalent in white females across all age groups, and the age of first ICD-10 encounter diagnosis was heavily skewed towards the 0–30 age cohort with a prevalence of 259.6 per 100,000. This data shows that EDS is frequently diagnosed in the early decades of life. As many included ocular ICD-10 diagnoses increase with age, the prevalence and risk in EDS patients may be underestimated compared to age-matched comparators.

Yet, many of the included ocular diagnoses were more prevalent in EDS records, especially among patients 0–60 years old. Overall, patients diagnosed with EDS had the highest prevalence of ICD-10 diagnoses for myopia (prevalence in EDS per 100,000: 5227.0), dry eye syndrome (4211.6), IIH (1995.4), and glaucoma (1532.8). Patients diagnosed with EDS had the highest PORs for diagnoses of angioid streaks (POR 18.72, CI 10.32, 33.94, *p* value 0) and IIH (POR 18.43, CI 17.51, 19.39, *p* value 0), while having decreased odds for diagnoses of T2 DR, AMD, and hypertensive retinopathy. Across every EDS age group, IIH consistently demonstrated the highest increased odds, and carotid cavernous fistula emerged as one of the most significant conditions in patients aged 0–30 (POR 17.21, CI 14.18, 20.88, *p* value 0).

Our data suggests that an EDS diagnosis has significant implications on ocular health. The altered mechanisms of procollagen formation, extracellular matrix (ECM) bridging molecules, and glycosaminoglycan synthesis contribute to the complex pathogenesis of EDS and its subtypes [27]. Collagenous structures of the eye include the cornea, sclera, vitreous body, lens capsule, retina, and choroid [28, 29]. Thus, these widespread ocular pathologies with demonstrated increased odds in EDS records confirm that EDS collagen malformations extend systemically and a referral to ophthalmology should not be undermined.

Confirming smaller studies on ocular manifestations of EDS, we found increased odds of ICD-10 diagnoses for IIH (overall POR 18.43), myopia (4.40), degenerative myopia (3.48), carotid cavernous fistula (3.99), and dry eye (3.89) in both our overall and age-stratified patient groups (Fig. 1a–d). Although angioid streaks had a markedly elevated POR (18.72) in the overall group, there were only 11 EDS records with angioid streaks. Additionally, there exist controversial associations between angioid streaks and EDS in the literature which can introduce sources of misdiagnosis and an inadvertent inflation of its true prevalence rate in this population [30–32]. Combined with the rarity of this diagnosis, the POR for angioid streaks may be inadvertently skewed, making this association an area for future study.

Myopia and degenerative myopia are two of the most commonly reported ocular findings in EDS, yet the literature remains inconclusive with contradictory findings [1, 18, 33]. Our findings show that patients diagnosed with EDS have a higher POR of myopia compared to the general population. Myopia is typically diagnosed in childhood, and adult-onset myopia progression can be observed up to the 5^th^ decade of life [34]. Given that the mean age of EDS records was higher than its general comparator in the 0–30 age cohort, when a myopia diagnosis is most common, the impact of our findings is particularly pronounced. The proposed pathogenesis involves EDS mediated changes to the vitreous ECM secondary to decreased bridging molecule integrity and altered scleral composition secondary to abnormal collagen formation. Both ECM and scleral structure regulate the axial length of the eye, and therefore EDS impacts on both of these components can lead to myopia and its progressively degenerative subtypes [18, 35]. Additionally, animal studies examining EDS mouse models have shown abnormal collagen structure in the cornea, causing changes to the biomechanical properties of the cornea [36, 37]. Others have also found that classic EDS patients have both thinner and steeper corneas [19, 38–40]. Such corneal changes may also explain the pathophysiology of increased myopia seen in EDS.

Villani et al. and Gharbiya et al. hypothesize over the role of EDS in dry eye syndrome, which we also found to be increased in patients diagnosed with EDS. Increased ocular surface disease index scores and tear film instability in EDS patients suggests a role in the altered collagen of the ocular surface epithelial cells [19, 41]. Alternatively, altered ECM of the lacrimal glands may explain the association between EDS and dry eyes [18].

The increased odds of an IIH diagnosis in EDS may be due to altered dynamics of cerebrospinal fluid secondary to Chiari malformation comorbid with hypermobile EDS [22, 42, 43]. Inferior displacement of the cerebellar tonsils creates an elongated and obstructed system for cerebrospinal fluid outflow which promotes a hypertensive state in the subarachnoid space, directly affecting the optic nerve and causing IIH findings such as papilledema [44, 45]. Another hypothesis is related to the increased odds of finding carotid-cavernous fistula formation in vascular EDS patients [46, 47]. Pollack et al., reports that structural deficits in type III collagen have been associated with ophthalmic arterial wall abnormalities and concurrent fistula formation with the cavernous sinus [48]. Resulting changes to cavernous sinus pressure may act as a nidus for turbulent flow and increased pressure around the optic nerve [49].

Conversely, T2 DR, AMD, and hypertensive retinopathy were among those diagnoses with the most significantly decreased odds in overall patients diagnosed with EDS. This may be the combination of the shortened life expectancy of people with EDS (median life span of 48 years), younger mean ages of the EDS cohort in the dataset, and the delayed presentation of T2 DR, AMD, and hypertensive retinopathy leading to diagnoses at later stages of life [50–52]. This is further corroborated by the dramatically lower numbers of EDS diagnoses in the 61+ cohort and lack of significant PORs for the ocular conditions in this cohort (Fig. 1d). Future study pursuits could aim to include age-matching and explore the prevalence of the disease, such as diabetes, that underlies the related ocular comorbidity in EDS patients.

Another notable finding in this study was that a diagnosis of ROP had significantly decreased odds in EDS records. ROP is most often a consequence of preterm birth and pregnancies, and an EDS foetus has a higher rate of preterm birth [53–55]. The opposite finding in our study raises further questions about the pathogenesis of ROP with EDS that would benefit from study from a molecular perspective.

Limitations inherent to the use of the platform include lack of visibility into patient cases if the query results in patient counts between 1–10 patients. Thus, rare conditions, especially when broken into 30-year-age cohorts, could not be included in the study to preserve the accuracy of the analysis. Additionally, because the platform depends on accurate diagnoses and reliable ICD coding, the study data is likely an underestimation of true clinical cases. The platform’s general population carries an inherent bias towards those utilizing the healthcare system, as it captures anyone who has a visit recorded in the EHR. Thus, although it is not a true measure of the US population, the platform has been found to have comparable percentages of patients to the US Census with the exception of Hispanic patients [56]. Similarly, because socioeconomic factors cannot be coded for, uncontrollable confounding variables may have been introduced into the study. A limitation inherent to the study was the inability to gather nor analyse data on EDS subtypes because the platform did not contain this granularity. A final study limitation involves bypassing Bonferroni correction through the statistical analysis of multiple comparisons on the dataset. This was attempted to be addressed through the inclusion of the *p* values in the figures.

In conclusion, this study demonstrated that an EDS diagnosis was most prevalent in white females aged 0–30 years old. Additionally, it showed that patients aged 0–60 years old who are diagnosed with EDS are at increased odds for many ocular conditions that involve collagenous structures of the eye, compared to counterparts of the same age. Specifically, diagnoses of IIH, myopia, dry eye syndrome, and carotid cavernous fistulas were among the highest odds in EDS records. Although angioid streaks demonstrated markedly increased odds in EDS records, this condition was ultimately too rare to be studied in this platform and may be better suited for targeted retrospective study. However, the frequency of ocular findings in EDS patients suggests that an ophthalmological referral may be a beneficial addition to the initial care of a newly diagnosed EDS patient. Inclusion of screenings for these conditions can also guide patient-provider conversations on the potential impacts of an EDS diagnosis on the patient’s ocular health. Future studies should examine the effects of these findings after propensity matching by potential confounders such as age, further clarify the pathogenesis of EDS ocular manifestations, or explore the associations between ocular conditions and causes of EDS morbidity, such as cardiovascular events.

## Summary

### What was known before:

- Ehler-Danlos Syndrome (EDS) is a genetic disorder of collagen formation.
- Ocular manifestations are not well characterized, with literature largely consisting of case reports or case series which report a widely varied patient presentation.
- Collagen malformations are pervasive in the eye given the pervasiveness of ocular collagenous structures.

### What this study adds:

- Patients diagnosed with EDS are at increased odds of having a recorded diagnosis of a concurrent ocular pathology, notably idiopathic intracranial hypertension.
- Patients diagnosed with EDS are at decreased odds for ocular diagnoses with an older age at presentation, such as diabetic retinopathy or age-related macular degeneration.
- Ophthalmological workup, or referral to ophthalmology, should be considered in a patient diagnosed with EDS to monitor for the presence or progression of these ocular conditions

## Data Availability

The datasets generated during and/or analysed during the current study are available from the corresponding author on reasonable request.
